# Performance of Fully-Automated High-Throughput Plasma Biomarker Assays for Alzheimer's Disease in Amnestic Mild Cognitive Impairment Subjects

**DOI:** 10.14283/jpad.2024.58

**Published:** 2024-03-19

**Authors:** G.M. Giuffrè, Davide Quaranta, M.G. Vita, E.M. Costantini, S. Citro, C. Carrozza, G. De Ninno, P. Calabresi, C. Marra

**Affiliations:** 1 Neurology Unit Fondazione Policlinico Universitario Agostino Gemelli IRCCS, Rome, Italy; 2 Memory Clinic Fondazione Policlinico Universitario Agostino Gemelli IRCCS, Rome, Italy; 3 Department of Neuroscience, Catholic University of the Sacred Heart, Rome, Italy; 4 UOC of Chemistry, Biochemistry and Clinical Molecular Biology - Fondazione Policlinico Universitario Agostino Gemelli IRCCS, Rome, Italy; 5 Università Cattolica del Sacro Cuore, Fondazione Policlinico Universitario ‘A. Gemelli', Largo Agostino Gemelli 8, 00168, Rome, Italy

**Keywords:** Alzheimer's disease (AD), amnestic mild cognitive impairment (aMCI), blood-based biomarkers, plasma biomarkers, fully-automated assays

## Abstract

**Introduction:**

Novel plasma biomarkers are promising for identifying Alzheimer's disease (AD) pathological processes in vivo, but most currently employed assays have limitations precluding widespread use.

**Methods:**

CSF and plasma samples were collected from seventy amnestic mild cognitive impairment (aMCI) subjects, stratified as A+ and A−. CSF Aβ40, Aβ42, p-tau181 and t-tau and plasma Aβ40, Aβ42 and p-tau181 quantification were conducted using the Lumipulse G assays (Fujirebio), to evaluate the diagnostic performance of plasma biomarkers and assess their associations with CSF biomarkers.

**Results:**

All plasma biomarkers except Aβ40 showed a very good accuracy in distinguishing A+ aMCI from A− aMCI, Aβ42/p-tau181 ratio being the most accurate (AUC 0.895, sensitivity 95.1%, specificity 82.8%). Plasma biomarkers levels were significantly associated with CSF biomarkers concentration.

**Discussion:**

High-throughput and fully-automated plasma assays could be helpful in discriminating with high accuracy between aMCI in the AD continuum and aMCI unlikely due to AD in clinical settings.

## Background

Dementia caused by neurodegenerative diseases affects over 50 million people worldwide, Alzheimer's disease (AD) being the most common cause. As the average life expectancy increases, the prevalence of these diseases is expected to grow in the next decades, resulting in considerable social and economic burden (1, 2). AD is pathologically defined by two major hallmarks: the accumulation of amyloid-β (Aβ) in extracellular plaques and the aggregation of hyperphosphorylated tau (p-tau) in intracellular neurofibrillary tangles. Currently, established biomarkers such as those obtained by cerebrospinal fluid (CSF) assays and positron emission tomography (PET) imaging provide objective measures of these pathological processes during life, playing a crucial role in early disease detection and differential diagnosis (3). However, the limited availability, invasiveness, high costs, and contraindications associated with these methods hinder their widespread use. Because of this compelling need for cost-effective biomarkers that could be less invasively obtained, researchers have been exploring the development of ultra-sensitive detection methods for blood-based biomarkers.

Although the prospect of reliable assays for blood biomarkers of amyloidopathy and tauopathy has historically posed challenges, recently technological advancements have paved the way for the development of sensitive and specific analytical approaches. In 2016 plasma Aβ42/40 ratio assessed using a novel single molecule array (Simoa) immunoassay demonstrated moderate accuracy in detecting abnormal Aβ status (4), and subsequent studies using both mass spectrometry (MS) approaches (5-7) and immunoassays (8) confirmed that plasma Aβ42/40 can accurately identify AD pathology in vivo, with performance metrics that could rival the gold standards of Aβ-PET and CSF. While certain MS-based methods exhibited superior performances to immunoassays in a head-to-head comparison (9), their complexity and limited accessibility could limit their widespread application, while the use of fully-automated, high-throughput, and highly reliable immunoassays may facilitate the clinical implementation of blood-based biomarkers. However, relying solely on the Aβ42/40 ratio as a plasma biomarker for AD pathology encounters significant challenges due to its minimal reduction in A+ subjects compared to the reduction observed in CSF (7).

Recent years have also seen the development of several novel assays for quantifying different plasma p-tau epitopes. The strong association between plasma p-tau181 and Aβ-positivity has been demonstrated through various assays (10, 11, 12), and heightened attention has been directed towards other p-tau epitopes (most notably p-tau217), which have emerged as promising targets for detecting biologically defined AD with even greater diagnostic accuracy, as corroborated by research comparing different p-tau biomarkers within the same cohort (13, 14). Plasma p-tau181 concentrations measured using Simoa have also shown to accurately predict future progression to dementia in a mild cognitive impairment (MCI) population, and a certain degree of accuracy in distinguishing between Aβ-positive and Aβ-negative MCI subjects (AUC=0.79) (15). More recently, potential diagnostic and prognostic workflows based on combinations of multiple biomarkers and other accessible and cost-effective measures are being proposed (16, 17). An approach for predicting Aβ status in MCI subjects in clinical settings based on plasma p-tau217, age and APOE ε4 status followed by confirmatory lumbar punctures only in uncertain cases has achieved a high classification accuracy (ranging between 88.2 and 92%, depending on the chosen thresholds) and a substantial reduction in referrals for further confirmatory testing (18), showing a potential appropriate use of novel blood biomarkers as a first-line screening tool.

Nevertheless, the high costs and the specialized staff requirements of many instruments may still limit their widespread implementation in clinical practice. Moreover, several plasma biomarker assays rely on proprietary reagents or are for research use only, even if few novel immunoassays are currently being validated for clinical use in some countries (19). In light of these challenges, instruments allowing high-throughput, fully-automated, and reliable plasma biomarker assays are indeed the focus of current research. A recent study (20) reported the reliability of the new Lumipulse G p-tau181 plasma assays in differentiating AD from cognitively unimpaired A− subjects, suggesting the potential of this fully-automated instrument.

The aim of this cross-sectional study was to evaluate the diagnostic performance of the Lumipulse G plasma Aβ40, Aβ42 and p-tau181 assays in a group of amnestic mild cognitive impairment (aMCI) subjects and assess the associations between plasma and CSF biomarkers.

## Methods

Seventy subjects with a clinical diagnosis of aMCI according to current clinical criteria (21, 22), were consecutively enrolled among native Italian-speaking patients referring to the Neuropsychology Unity – Memory Clinic of the Policlinico A. Gemelli in Rome for diagnostic evaluation of memory loss and cognitive impairment. All subjects underwent a thorough clinical and neurological examination, as well as a standard extensive neuropsychological investigation for assessment of different cognitive domains, routine blood tests, and a standard brain magnetic resonance imaging. For the definition of aMCI, the following characteristics were considered: memory decline reported by an informed caregiver or by the subject; evidence of memory impairment on tasks assessing verbal or visuospatial episodic memory; preserved independence in activities of daily living. Exclusion criteria were as follows: Clinical Dementia Rating score >0,5; functional impact on activities of daily living; previous or concomitant neurological diseases; major psychiatric disorders; medical conditions potentially able to interfere with cognitive functions; and previous participation in experimental studies with amyloid-targeting agents. All subjects provided an informed written consent before undergoing lumbar puncture and gave their informed consent to the use of their clinical and biological data for anonymized studies.

CSF and blood samples were collected, stored, and processed according to literature-based (23, 24, 25, 26) standardized operating procedures. CSF was obtained through lumbar punctures performed in the morning after an overnight fast, using 22-gauge Quincke spinal needles, collected in polypropylene tubes, centrifuged within 1 hour at 2000x g for 10 min and then immediately aliquoted in polypropylene vials and frozen at −80°C. Blood was collected through venepunctures in EDTA-plasma tubes, centrifuged within 1 hour at 2000x g for 10 min and then aliquoted in polypropylene vials and stored at −80°C. Levels of CSF Aβ40, CSF Aβ42, CSF p-tau181 CSF t-tau, plasma Aβ40, plasma Aβ42 and plasma p-tau181 were measured using the Lumipulse G assays on the LUMIPULSE G600II instrument (Fujirebio), a fully-automated system based on a two-step sandwich chemiluminescent enzyme-immunoassay (CLEIA).

ATN status (3) was determined according to established CSF biomarkers cut-offs (27) and subjects were therefore grouped as A+ (aMCI due to AD) and A− (aMCI unlikely due to AD) based on the value of the CSF Aβ42/40 ratio, preferred over CSF Aβ42 as a direct marker of amyloid pathology.

Statistical analyses were performed using SPSS and R software. All p-values were two-tailed and significance level for all analyses was set at α=0.001. Comparisons between continuous variables were carried out using Mann-Whitney U-test, while the χ^2^ test was used to compare dichotomous variables. Logistic regression models were developed, using Aβ-status as the dependent variable and plasma biomarkers as predictors, including age and sex as covariates in the analyses. Correlations between CSF and plasma biomarkers values were assessed through Spearman's correlation. Receiver-operating-characteristic (ROC) curve analysis was performed to assess the performance of plasma biomarkers in discriminating between the two aMCI groups. Cut-offs for plasma biomarkers were estimated based on the optimal trade-off between sensitivity and specificity (Youden Index).

## Results

The study sample comprised 41 A+ aMCI (22 female; mean age: 68.46 ± 6.24 years; mean literacy 14.02 ± 2.90 years; mean MMSE score: 25.95 ± 1.88) and 29 A− aMCI (10 female; mean age: 67.86 ± 6.50 years; mean literacy 13.83 ± 4.00 years; mean MMSE score: 26.69 ± 2.39) subjects. The groups did not differ in terms of age (p=0.793), sex (p=0.113), literacy (p=0.711) and MMSE (p=0.125).

**Figure 1:**
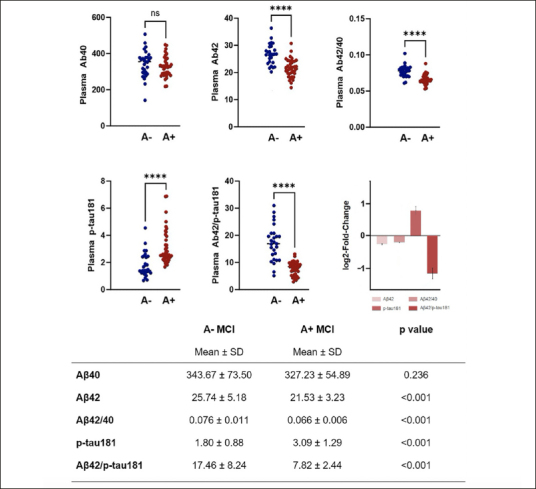
Plasma biomarkers values in aMCI due to AD and aMCI unlikely due to AD Plasma biomarkers values in aMCI due to AD and aMCI unlikely due to AD. Plasma biomarkers log2-fold-change values (with standard error) are also represented.

Values of plasma biomarkers measured using the Lumipulse G are reported in Figure 1. As expected, aMCI due to AD group showed significantly reduced levels of plasma Aβ42, Aβ42/40 ratio and Aβ42/p-tau181 ratio and significantly increased levels of p-tau181. No differences between groups were seen in relation to plasma Aβ40 levels.

In logistic regression analyses controlled for age and sex, plasma Aβ42 (B=−0.277, p=0.001), plasma Aβ42/40 ratio (B=−157.576, p<0.001), plasma p-tau181 (B=1.685, p<0.001) and plasma Aβ42/p-tau181 ((B=−0.459, p<0.001) were found to significantly predict Aβ-status determined by CSF examination.

**Figure 2:**
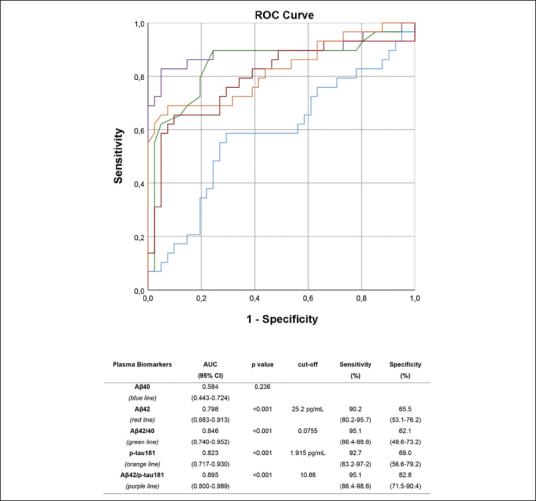
ROC curves ROC curves: performance of plasma Aβ40 (blue), Aβ42 (red), Aβ42/40 ratio (green), p-tau181 (orange) and Aβ42/p-tau181 (purple) in differentiating aMCI due to AD from aMCI unlikely due to AD. Optimal cut-offs maximizing sensitivity and specificity are reported.

ROC analyses were performed to assess the performance of plasma biomarkers and their ratios in differentiating between aMCI due to AD and aMCI unlikely due to AD (Figure 2). All plasma biomarkers except for Aβ40 showed a very good accuracy, with the Aβ42/p-tau181 ratio being the most accurate, with an AUC of 0.895 (CI 0.800–0.989), showing a sensitivity of 95.1%, a specificity of 82.8%, a NPV of 88.6%, and a PPV of 92.3%.

Regarding the correlations between plasma and CSF biomarkers, all examined associations were statistically significant, except for those involving CSF and plasma Aβ40 levels: plasma Aβ42, Aβ42/40 ratio, p-tau181 and Aβ42/p-tau181 ratio were significantly associated with CSF Aβ42 levels (ϱ=0.507, p<0.001; ϱ=0.471, p<0.001; ϱ=−0.480, p<0.001; ϱ=0.596, p<0.001), CSF Aβ42/Aβ40 ratio (ϱ=0.528, p<0.001; ϱ=0.520, p<0.001; ϱ=−0.454, p<0.001; ϱ=0.578, p<0.001), CSF p-tau181 concentration (ϱ=−0.562, p<0.001; ϱ=−0.484, p<0.001; ϱ=0.536, p<0.001; ϱ=−0.690, p<0.001), CSF total-tau concentration (ϱ=−0.421, p<0.001; ϱ=−0.410, p<0.001; ϱ=0.417, p<0.001; ϱ=−0.542, p<0.001), and CSF Aβ42/p-tau181 ratio (ϱ=0.599, p<0.001; ϱ=0.496, p<0.001; ϱ=−0.553, p<0.001; ϱ=0.697, p<0.001); while there were no statistically significant associations between plasma Aβ40 and any of the CSF biomarkers. Plasma Aβ42/pTau ratio had the strongest correlations with all CSF biomarkers.

## Discussion

The current study revealed that AD plasma biomarkers measured by the new Lumipulse G plasma assays are quite effective in distinguishing between aMCI in the AD continuum and aMCI unlikely due to AD. Using the Aβ42/p-tau181 ratio could identify A+ aMCI in our population with high accuracy (89.5%) and sensitivity (95.1%). Moreover, plasma biomarkers values were found to be significantly associated with CSF biomarkers concentration.

Early works with conventional assays did not yield definitive conclusions regarding changes in plasma biomarker levels in AD. Recently, new high-sensitive techniques have been developed, with pros and cons in terms of technical capacities and clinical translatability. Indeed, although MS-based methods offer high accuracy (9, 13), their complicated nature, high costs, expensive maintenance, and limited availability in general hospitals are currently not compatible with routine clinical use (19). On the other hand, novel immunoassay techniques have reached tremendous improvements in terms of analytical sensitivity (28, 29), and the availability of fully-automated instruments such as the Lumipulse G would facilitate widespread clinical application, due to their accurate quantitative results with high sensitivity and reproducibility, low intra- and inter-assay variability, easy handling and high throughput. This platform has already been well validated for CSF biomarkers (27) and has the potential for making the biological diagnosis of AD generally accessible beyond specialized centres with access to PET or CSF biomarkers. Previous research on Lumipulse G p-tau181 plasma assays (20) compared patients with AD dementia to A− clinically unimpaired individuals, maximizing the contrast between groups and obtaining a high accuracy. In the present study we selected a homogenous sample of aMCI subjects, which poses a greater challenge in achieving a high diagnostic accuracy. In this clinical setting, the diagnostic value of this fully-automated system seems high, which could be relevant since subjects with prodromal AD are likely to be the main target population for disease-modifying therapies. Implementing plasma biomarkers in clinical practice could significantly reduce the burdens and costs associated with the exams currently used to identify subjects in the AD continuum, facilitating accurate diagnosis, supporting appropriate patient selection for disease-modifying therapy, and enhancing the detection of drug and lifestyle interventions effects on disease progression (30). Moreover, it could pave the way for the development of cost-effective screening methods to be used in real-life population studies. For these reasons, plasma biomarkers will be incorporated into the revised criteria for biomarker categorization, disease diagnosis and staging of Alzheimer's disease proposed by the Alzheimer's Association workgroup, with the potential for fully-automated high-throughput plasma biomarker assays to revolutionize both clinical care and research.

Although the cutoff values herein evaluated have demonstrated good sensitivity and specificity, it is crucial to acknowledge that establishing a universal binary threshold to impose a clear-cut separation between negative and positive subjects (as done for CSF biomarkers) may not be the most appropriate choice for blood biomarkers. Opting for the use of diverse cutoffs tailored to the intended uses - whether to prioritize high sensitivity for developing first-line screening programs or employing multiple risk thresholds to identify individuals at high, intermediate, and low risk of AD – might be more fitting (17). Moreover, incorporating combinations between novel blood biomarkers and other accessible and cost-effective biomarkers (such as cognitive tests and APOE genotyping) will be essential for the elaboration of both diagnostic and prognostic algorithms.

The present study has some limitations. Despite the robust results provided on a cohort of well-characterized aMCI subjects, the sample was relatively small, and data were obtained from a single site. Although the employed instrument is fully-automated, future studies will be essential to validate these findings in multi-centric studies on larger cohorts. Furthermore, our study lacked other plasma p-tau epitopes, such as p-tau231 and p-tau217. Notably, the latter is currently emerging as one of the most promising biomarkers for AD pathology (13, 14) and, therefore, may deserve further investigation in the aMCI population in the future. Moreover, the lack of longitudinal data does not allow to assess the associations between plasma biomarkers and the trajectory of cognitive decline.

To conclude, Lumipulse G plasma biomarker assays demonstrated a high degree of accuracy in discriminating between aMCI in the AD continuum and Aβ-negative aMCI, also showing a robust association with corresponding CSF biomarkers. This study expands the growing body of research on AD plasma biomarkers and exhibits the diagnostic value of a promising fully-automated technology that could enable widespread implementation of cost-effective and easily accessible blood-based biomarkers, simplifying and accelerating the diagnostic process for subjects with cognitive decline.

*Funding:* This research did not receive any specific grant from funding agencies in the public, commercial, or not-for-profit sectors. Open access funding provided by Università Cattolica del Sacro Cuore within the CRUI-CARE Agreement.

*Ethical standards:* Ethical approval was given by the local ethical committee at Fondazione Policlinico Agostino Gemelli IRCCS, and the research was conducted in accordance with the Helsinki Declaration.

*Conflict of interest:* On behalf of all authors, the corresponding author states that there is no conflict of interest.
